# Individualized intravenous thrombolytic strategy for acute ischemic stroke with large vessel occlusion on the era of mechanical thrombectomy: cases report

**DOI:** 10.1007/s10072-019-04098-6

**Published:** 2019-11-14

**Authors:** Pengfei Xing, Hongjian Shen, Zifu Li, Pengfei Yang, Yongwei Zhang, Jianmin Liu

**Affiliations:** Department of Cerebrovascular Disease Center, Changhai Hospital, Second Military Medical University, No.168 Changhai Rd, Shanghai, 200433 China

**Keywords:** Intravenous thrombolysis, Mechanical thrombectomy, Ischemic stroke

## Abstract

Intravenous thrombolysis for acute ischemic stroke within 4.5 h after the onset of symptoms has become a standard therapy that is recommended by many trials and clinical guidelines. As on the era of mechanical thrombectomy for acute ischemic stroke with large vessel occlusions, whether intravenous thrombolysis (IVT) is still necessary, and how to choose the optimal dose are still controversy. Here, we reported two cases of acute ischemic stroke with large vessel occlusions that both achieved complete recanalization after IVT. Then, IVT was terminated in advance, and dynamic surveillance by DSA was performed to achieve individual treatment. However, both of the cases presented with hemorrhagic transformation. We analyzed the probable reasons and put forward thoughts from ourselves.

## Introduction

Intravenous thrombolysis (IVT) for acute ischemic stroke within 4.5 h after the onset of symptoms, using recombinant tissue-type plasminogen activator (rtPA) with the recommended dose of 0.9 mg/kg, is a standard therapy and recommended by various trials and guidelines [1–3]. However, the appropriate dose is still being controversial considering the balance of effectiveness and safety. Some studies showed that low-dose rtPA was comparable with the standard-dose treatment in terms of effectiveness [4–7]. In contrast, someone indicated that standard-dose intravenous rtPA could achieve more favorable outcome without increasing the risk of symptomatic hemorrhage than low-dose rtPA [8]. Moreover, comparing with IVT, mechanical thrombectomy (MT) has been proved to be more effective for acute ischemic stroke with large vessel occlusion (AIS-LVO) due to the high rates of successful recanalization (modified TICI 2b–3 grades) [9–15]. The subgroup study of HERMES collaboration suggested that the effects favoring the intervention was significant in those not receiving intravenous alteplase [16]. Therefore, the major controversy is that whether it is necessary to perform IVT before MT for AIS-LVO within the thrombolytic time window. We reported two cases of AIS-LVO that was treated by only IVT, and the dose of intravenous rtPA was controlled individually according to their timely angiography results.

### Case 1

**Fig 1:**
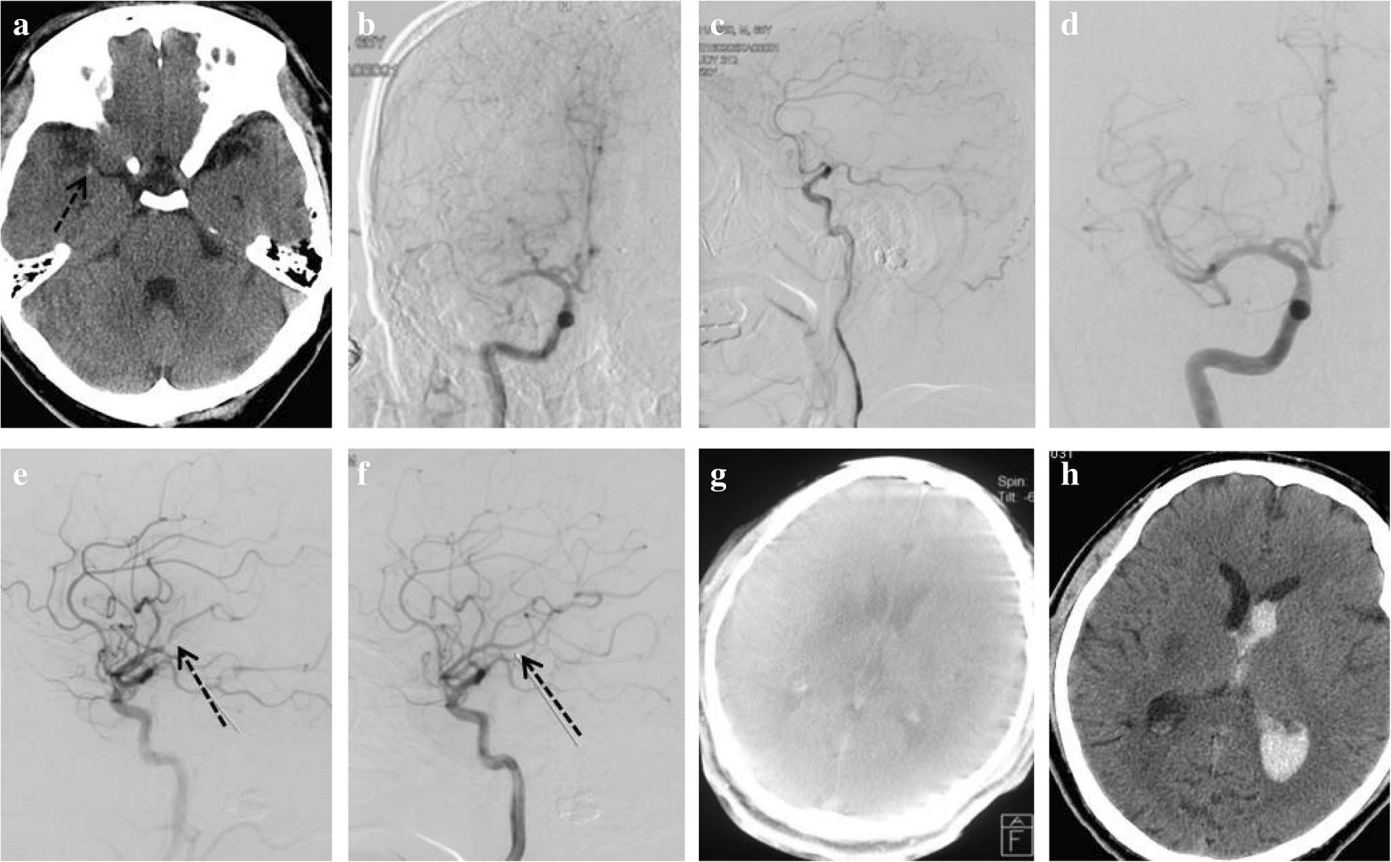
Images of case 1. **a** Non-contrast CT: a hyperdense right MCA (black arrow). **b** Cerebral angiography: occlusion of the M1 segment of right MCA from frontal image. **c** Cerebral angiography: occlusion of the M1 segment of right MCA from lateral image. **d** Cerebral angiography: spontaneous recanalization of the M1 segment of right MCA before endovascular intervention. **e** Cerebral angiography: thrombus moving to MCA bifurcation 5 min later (black arrow). **f** Cerebral angiography: thrombus disappeared (black arrow) and complete recanalization of the right MCA. **g** Dyna-CT image: no hemorrhagic transformation. **h** Follow-up non-contrast CT at 24 h after IVT: right thalamus hemorrhage

A 67-year-old male presented with sudden left limbs deficit was admitted to our hospital at 33 min after symptom onset. Non-contrast head CT was performed immediately (multimodal CT scan was not available at that time) and revealed no intracranial hemorrhage but with high- density on the right middle cerebral artery (MCA) M1 segment (Fig. 1a). The National Institute of Health Stroke Scale (NIHSS) scored 14, and the patient was treated with IVT (rtPA 0.9 mg/kg × 78 kg) that followed by digital subtraction angiography (DSA). The first image of DSA showed that the right MCA M1 segment was occluded with a score of American Society of Interventional and Therapeutic Neuroradiology collateral grading (ACG) system 3 (Fig. 1b and c). However, the M1 segment was recanalization 6 min after the first DSA image, and the residual thrombus had immigrated to the M2 segment (Fig. 1d and e). Fortunately, the vessel achieved complete recanalization after another 5 min without any endovascular treatment in the whole process (Fig. 1f). Therefore, IVT was terminated in advance with a total dose of 42 mg (3/5 standard-dose, 0.54 mg/kg individually). The door-to-needle time (DNT), door-to-puncture time, onset-to-needle time, and onset-to-reperfusion time were 30 min, 58 min, 63 min, and 106 min, respectively. Immediate result of Dyna-CT scan (Artis zee biplane, Siemens, Germany) confirmed no intracranial hemorrhage (Fig. 1g), and the NIHSS score decreased to 1 at 24 h. However, asymptomatic intracranial hemorrhage in the infarction area was detected by the non-contrast CT imaging at 24 h, with class 3 of the Heidelberg Bleeding Classification [17] (Fig. 1h). Finally, the modified Rankin Score (mRS) of the patient at 90 days was 0.

### Case 2

**Fig 2.**
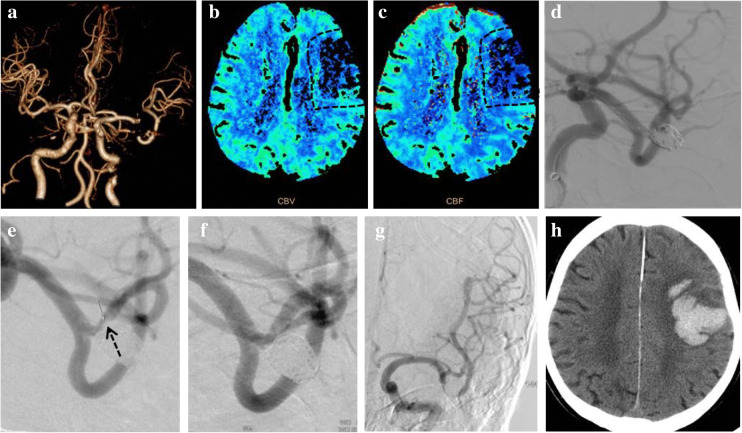
Images of case 2. **a** CT angiography: occlusion of the M1 upper branch of left MCA (white arrow). **b** CT perfusion: map of cerebral blood volume revealing low volume in the left frontal and parietal lobe (white dotted frame). **c** CT perfusion: map of cerebral blood flow revealing decreased flow in the left frontal and parietal lobe (white dotted frame). **d** Cerebral angiography: partial recanalization of the M1 upper branch with recovering forward flow and coil embolization assisted with stent for unruptured bifurcation aneurysm. **e** Cerebral angiography: residual thrombus in M1 bifurcation (black arrow). **f** Cerebral angiography: thrombus disappeared completely. **g** Cerebral angiography: complete recanalization of the upper branch. **h** Follow-up non-contrast CT at 24 h after IVT: left frontal and parietal hematoma

An 80-year-old male presented with sudden slurred speech and weakness of the right limbs was sent to our hospital at 102 min after the symptom onset. The pre-procedure NIHSS score was 14 and a multimodal CT was performed. There was no intracranial hemorrhage and significant infarcted territory on non-contrast CT imaging. However, the CT angiography showed occlusion of the upper branch of left MCA M1 segment with lower perfusion that was revealed by cerebral blood volume and cerebral blood flow on CT perfusion (Fig. 2a–c). The patient received stenting of the responsible artery to assist coil embolization of an unruptured bifurcation aneurysm 2 months ago (Fig. 2d) and took aspirin (Bayer, Germany) 100 mg/day before stroke. As the patient was still within the thrombolytic time window, we administered rtPA (0.9 mg/kg × 60 kg) and performed DSA immediately. The DSA showed that the part of forward blood flow had recovered, but thrombus was still on the ostium of the artery (Fig. 2e). Then, IVT was continued and the occluded artery was recanalized to modified thrombolysis in cerebral infarction (mTICI) 3 (Fig. 2f and g) 15 min later without any endovascular intervention. Then, we stopped IVT when the total dose of rtPA was 45 mg (5/6 standard dose, 0.75 mg/kg individually). But the symptom was not improved, and the NIHSS score was still 14. The DNT, door-to-puncture time, onset-to-needle time, and onset-to-reperfusion time were 35 min, 63 min, 137 min, and 182 min, respectively. Non-contrast CT imaging showed hemorrhagic transformation (HT) with class 2 of the Heidelberg Bleeding Classification and PH2 of ECASS II [18] at 24 h (Fig. 2h). The location of HT was similar with the lower perfusion territory on the CT perfusion scan before IVT. Although the intracranial hemorrhage was absorbed within 1 week, clinical symptoms were not recovered, and the mRS was 4 at 90 days.

## Discussion

Here, we reported two cases of acute ischemic stroke with large vessel occlusions that both achieved complete recanalization after IVT. Then, IVT was terminated in advance, and dynamic surveillance by DSA was performed to achieve individual treatment. However, both of the cases were presented with hemorrhagic transformation. We analyzed the probable reasons and put forward thoughts from ourselves.

Both patients were Asian. The first patient didn’t receive any antiplatelet or anticoagulation treatment before IVT, while the second patient received aspirin to prevent intra-stent thrombosis after stent-assisted embolization of aneurysm. Previous studies indicated that Asian populations had a higher prevalence of cerebral hemorrhage due to prior antiplatelet therapy, elderly patients, diabetes, and/or uncontrolled hypertension [19–22]. Therefore, in these populations, low-dose rtPA might be preferred, and we should think about terminating IVT in patients with high-risk of HT.

The recommended dose of intravenous rtPA was 0.9 mg/kg according to the NINDS trial, and this dose is still preferred nowadays [1]. Considering the risk of intracranial hemorrhage, some trials had attempted to determine the right rtPA dose that could balance both effectiveness and safety, but obtained different results [4–8]. The majority of the thrombus had been lysed before angiography in 11% of the cases in the endovascular therapy group of the EXTEND-IA trial [12], which indicated that IVT was necessary for AIS within thrombolytic time window. However, the thought whether IVT should be stopped when angiographic recanalization to mTICI 3 was unknown. In our cases, the occluded arteries achieved completed recanalization under the dynamic surveillance of DSA, and we stopped the IVT early. However, the strategy failed to prevent HT associated with IVT in both cases.

We hypothesized that individual sensitivity to rtPA was responsible to HT outside the infarcted region rather than the dose of rtPA in the first case. We could not imagine what would happen if we had administered the whole standard-dose after complete recanalization was observed. Some studies showed that there was no correlation between the rtPA dose and HT [4, 23, 24]. However, there were opposite opinions [18, 25]. Studies proved that HT was associated with blood-brain barrier disruption due to reperfusion, and this correlation was stronger in patients receiving endovascular treatment than in patients receiving intravenous rtPA [26, 27].

Comparing with rtPA, successful reperfusion might be the major factor that was responsible for HT in the second case. There was a significant low perfusion region before IVT from perfusion images both on the cerebral blood volume and cerebral blood flow, considered as core infarction. The following non-contrast CT imaging had proved that HT just occurred in the infarct region before IVT. According to the excluded criteria of the published trials, this case was not suitable for endovascular treatment because of less benefit from the vascular recanalization due to a large core infarct territory [13–15]. IVT for this case might be harmful because of large core infarction. However, large core infarction was not an exclusive criteria for rtPA, and we had to perform IVT for the case following the guideline. We have no answers on whether it was better to choose regular antiplatelet therapy rather than recanalization using IVT or endovascular treatment for patients with large core infarction. We do not know whether it would decrease the rate of HT after IVT on the base of multimodal imaging. At least, so far, IVT within 4.5 h is still the recommended standard treatment for AIS and not limited by multimodal imaging.

Magnetic resonance imaging and computed tomography angiography could help to detect cerebral infarcts and occlusion head-neck vessels, but both of them are not recommended as exclusive criteria for IVT [2, 28, 29]. Nevertheless, magnetic resonance perfusion and CT perfusion were recommended as inclusive criteria for MT or IVT for patients with uncertain time or long time from onset to arriving hospital [12, 14, 30–32]. A meta-analysis proved that patients with ischemic stroke within 4·5–9 h from stroke onset or wake-up stroke with salvageable brain tissue who were treated with rtPA achieved better functional outcomes than placebo group [32].

If we had performed MT directly without IVT for the first case, would we save the patient from HT? Although there were no randomized trials comparing MT after IVT with MT alone in thrombolytic time window, a meta-analysis indicated that patients with MT after IVT had better functional outcomes, lower mortality, higher rate of successful recanalization, and equal odds of symptomatic intracerebral hemorrhage compared with MT alone [33]. However, the post hoc analysis of ASTER trial showed that there were no significant differences between IVT before MT and MT alone in 90 days favorable functional outcome, successful reperfusion rate, or hemorrhagic complication rate, excepting 90 days mortality [34]. Randomized controlled trials should be performed to answer it, such as DIRECT-MT [35] trial, which had completed recruiting.

The major limitation of this study was the case reported with lower level evidence. Further work will be performed to strengthen the result.

## Conclusion

IVT is still the standard treatment for AIS patients within 4.5 h from symptom onset. The individual dose of rtPA and treatment strategies for AIS-LVO on the era of MT still need more studies to demonstrate.
